# Protective Role of Electroacupuncture Against Cognitive Impairment in Neurological Diseases

**DOI:** 10.2174/1570159X22999240209102116

**Published:** 2024-02-12

**Authors:** Yueyang Xin, Siqi Zhou, Tiantian Chu, Yaqun Zhou, Aijun Xu

**Affiliations:** 1 Department of Anesthesiology, Hubei Key Laboratory of Geriatric Anesthesia and Perioperative Brain Health, and Wuhan Clinical Research Center for Geriatric Anesthesia, Tongji Hospital, Tongji Medical College, Huazhong University of Science and Technology, Wuhan, China

**Keywords:** Electroacupuncture, cognitive impairment, neurological diseases, Alzheimer's disease, vascular cognitive impairment, mechanisms

## Abstract

Many neurological diseases can lead to cognitive impairment in patients, which includes dementia and mild cognitive impairment and thus create a heavy burden both to their families and public health. Due to the limited effectiveness of medications in treating cognitive impairment, it is imperative to develop alternative treatments. Electroacupuncture (EA), a required method for Traditional Chinese Medicine, has the potential treatment of cognitive impairment. However, the molecular mechanisms involved have not been fully elucidated. Considering the current research status, preclinical literature published within the ten years until October 2022 was systematically searched through PubMed, Web of Science, MEDLINE, Ovid, and Embase. By reading the titles and abstracts, a total of 56 studies were initially included. It is concluded that EA can effectively ameliorate cognitive impairment in preclinical research of neurological diseases and induce potentially beneficial changes in molecular pathways, including Alzheimer’s disease, vascular cognitive impairment, chronic pain, and Parkinson’s disease. Moreover, EA exerts beneficial effects through the same or diverse mechanisms for different disease types, including but not limited to neuroinflammation, neuronal apoptosis, neurogenesis, synaptic plasticity, and autophagy. However, these findings raise further questions that need to be elucidated. Overall, EA therapy for cognitive impairment is an area with great promise, even though more research regarding its detailed mechanisms is warranted.

## 1 INTRODUCTION

Cognitive impairment refers to the dysfunction in one or more aspects of memory, language, visual space, execution, computation, comprehension, and judgment, which includes dementia and mild cognitive impairment (MCI) [1, 2]. MCI is a syndrome defined as a cognitive impairment that exceeds what should be expected for an individual’s age and education level but does not significantly affect activities of daily living [3]. Nonetheless, dementia refers to complex cognitive decline covering chronic or progressive dysfunction of cortical and subcortical function and can interfere with patients’ family, occupational, or social functioning [4, 5]. Many neurological diseases can lead to cognitive impairment, such as Alzheimer’s disease (AD) [6], stroke [7], chronic pain [8], and Parkinson's disease (PD) [9]. Cognitive impairment is often accompanied by some neuropsychiatric symptoms and decreased ability to perform daily living, negatively affecting patients’ and caregivers’ quality of life [1]. Besides, it can bring substantial emotional and economic burdens for patients and their families [10]. The decline of cognitive function caused by aging and the increasing prominence of demographic aging will bring dire challenges to public health and continue contributing to the global burden of disease [4, 11-13]. Therefore, treatment for cognitive impairment is crucial and necessary. Unfortunately, the currently available drugs for cognitive impairment are limited; the main drugs for dementia are cholinesterase inhibitors and memantine. Other medications include citicoline, calcium channel blockers, propentofylline, naftidrofuryl, huperzine, sertraline, and vinpocetine cilostazol are used in vascular dementia (VaD) [14, 15]. No medications have proven effective for MCI [16]; similarly, no evidence supports any drug intervention to prevent dementia [15]. Lifestyle modifications such as the Mediterranean diet and exercise are associated with protection against cognitive impairment and dementia [17-19]. Consequently, the development of new substantial treatment or preventive methods that could delay or prevent cognitive impairment is still urgent.

As one of the essential treatments in traditional Chinese medicine (TCM), acupuncture has a history of thousands of years. One of the theoretical bases of acupuncture includes the “qi” and “meridian” in the body. “Qi” refers to the vital energy in our body and flows in the specific channels: “meridian”, which causes diseases when there is an interruption in this flow [20]. The other basic premise is that there are hundreds of acupoints distributed throughout the body. Acupuncture needles can activate them to regulate body functions and improve the quality of life [21, 22] when patients or acupuncturists feel soreness, numbness, fullness, or heaviness, which we called De Qi or achieve Qi. These acupoints are fully activated [23]. Unlike manual acupuncture (MA), which requires inserting a thin metal needle into an acupoint and manually operating, electroacupuncture (EA) is a comparatively new method and its application is also very convenient. Electronic instruments stimulate the acupoint and transmit electrical signals to achieve the purpose of treatment. Compared with MA, EA has better consistency and repeatability in scientific studies [24, 25]. In recent years, some clinical trials indicated that EA was a potential therapeutic method for cognitive impairment [26-29]. However, the mechanism of EA alleviating cognitive impairment remains elusive. This review aims to discuss the therapeutic approach of EA against cognitive impairment in neurological diseases such as AD, PD, and stroke in preclinical studies. It is hoped that our work could arouse more interest in exploring the mechanisms and effects of EA on cognitive impairment caused by neurological diseases.

## 2 METHODS

We used a retrospective analysis of published articles in PubMed, Web of Science, MEDLINE, Ovid, and Embase. Only papers in English were evaluated further. Considering the current research status, we considered preclinical literature published within the ten years until October 2022. The search term included a combination of the following terms: electroacupuncture, electro-acupuncture, EA, cognitive impairment, mild cognitive impairment, cognition, and dementia. We only included preclinical studies related to neurological disorders. By reading the titles and abstracts, a total of 56 studies were initially included. Adobe illustrator (version: 2020, CA, USA), Adobe Photoshop (version: 2020, CA, USA), and Figdraw (www.figdraw.com) were used for drawing figures.

## 3 ALZHEIMER'S DISEASE

AD is the single most prominent reason for dementia, accounting for 50-75%, and is a condition that primarily occurs in later life, with prevalence doubling approximately every five years after age 65 [30]. Clinically, AD is characterized by progressive memory loss and cognitive decline, leading to early death years after diagnosis [31]. The significant pathological features of AD are amyloid-β peptide (Aβ) deposition plaques and neurofibrillary tangles (NFTs) [32]. From the perspective of TCM, the occurrence of AD is related to the decline in the functions of the five internal organs and the deficiency of the sea of marrow. It is characterized by the deficiency in origin and the excess in superficiality. The main aspect of the deficiency in origin lies in the kidney marrow deficiency and the deficiency of both qi and blood. The manifestation of the excess in superficiality lies in the turbid phlegm blocking the clear orifices, which leads to blood stasis blocking the brain. These two factors interact with each other, forming a vicious cycle. However, the cause of AD is still unclear.

In short, there are three types of AD models: spontaneous, interventional, and genetically modified. Interventional models represent chemicals injected into brain tissue or specific brain areas. Since AD does not occur naturally in rodents, transgenic strains, including 5XFAD, 3XTG-AD, and APP + PS1, are widely used for research. Besides, the senescence-accelerated mouse/prone (SAMP) model is also used for AD studies [33-35]. A brief literature summary about EA improving cognitive impairment in the AD model is presented in Table **1**. It is concluded that *baihui* (GV20) is the most commonly selected acupoint. GV20 belongs to the Du meridian (the government vessel), mainly distributed over the head, face, and along the midline of the back, and reflects yang in our body. Due to its role in clearing the mind, lifting the spirits, and promoting resuscitation, GV20 is technically used in neurological and psychiatric diseases [36]. Based on the Chinese medicine theory, GV20 combined with *shenshu* (BL23) can nourish kidney-essence and modify the Governor Vessel [37], which targets the Kidney marrow deficiency pattern in the pathogenesis of AD. Meanwhile, acupuncturing at other GV20-centered acupoint combinations, such as *dazhui* (GV14) and *shuigou* (GV26), also improves cognitive function [37]. *Shenting* (GV24), like GV20, is recommended as one of the principal acupoints for treating cognitive impairment in the expert consensus survey [38]. Besides, *zusanli* (ST36) has anti-inflammatory properties and mitigates working memory as well [39, 40]. Most studies in this section will refer to the above principles for acupoint formation. A few acupoints not mentioned above, including *yintang* (GV29) and *benshen* (GB13), can calm the mind and be used for adjuvant therapy based on the TCM theory. *Sanyinjiao* (SP6) and *neiguan* (PC6) are combined with other acupoints to improve cognitive function in patients [41, 42]. *Taixi* (KI3) also has the effect of opening the orifices and tonifying the kidney, which is often used in MCI [43]. There is no doubt that these acupoints can be used to select and rationally match in order to play a role in the treatment of cognitive impairment. The schematic diagram of acupoints is shown in Fig. (**1**).

### 3.1 Effect of EA on Cognitive Function in AD Models

The object recognition test, variants, and the Morris water maze are widely used in many animal models to assess learning and memory for behavioral tasks [44]. The learning process for the above tests involves the integrity of the hippocampus and related areas, as well as the connections of the prefrontal cortex [44, 45]. Morris water maze targets episodic-like learning and memory, and the object recognition test aims at recognition memory [44, 45]. In addition, the Y-maze test, used extensively, is a relatively simple maze test to assess spatial working memory [46].

In this part, all but two of the studies in this section used water mazes to test the cognitive function after EA treatment [47-72], some of which were further evaluated with new object recognition and (or) the Y-maze test [53, 65, 66, 71, 72]. Several behavioral tests that are less mentioned in this section include fear conditioning [70], to evaluate nondeclarative associative learning [73], location discrimination task [74], a particularly conducive behavioral test in animal models of hippocampal, to assess memory for location [75], and step-down avoidance test [55], also to assess memory impairment [76]. In general, EA can improve cognitive function in AD animal models regardless of the behavioral tests used. The mechanisms that may be involved will be further discussed.

### 3.2 Effect of EA on Aβ and NFTs

Aβ is a major component of AD-related amyloid plaques and is derived from the complex cleavage of amyloid-β protein precursor (APP), which is a type I single-pass transmembrane protein in humans consisting of 639 to 770 amino acids; it is highly expressed in the central nervous system (CNS) and plays a variety of physiological functions [77, 78]. APP processing largely relies on three proteolytic secretase enzymes: α-, β- and γ-secretase [77]. First, APP can change into soluble and carboxyterminal fragments (CTFs) cleaved by α or β-secretase; specifically, α-secretase leads to the formation of soluble amyloid precursor proteins (sAPP)α and CTF83. Meanwhile, β-secretase generates sAPPβ and CTF99. Then CTF99 cleavage by γ-secretase can generate Aβ, based on the γ-secretase site of cutting, different isoforms of Aβ, including Aβ37, 38, 39, 40, 42, and 43, will be generated. Aβ40 and Aβ42 are the two major isoforms in the brain [77, 79]. Aβ exerts a role to interfere with CNS through synaptic dysfunction, memory loss, damage to the brain structure, and a potent promoter of neuroinflammation [80, 81].

Tau protein is a highly enriched protein in neurons, initially defined as having the ability to bind and stabilize microtubules. However, its function has exceeded the regulation of microtubule dynamics and involves synaptic structure and function, mediating axonal transport and neuronal signaling pathways [82]. The balance between tau protein kinase and phosphatase regulates the phosphorylation state of tau. When tau is abnormally phosphorylated, it weakens its ability to bind to microtubules and causes self-assembly into tangles/fibers [82, 83]. NFTs are aggregated by tau, which exhibits hyperphosphorylation pathologically at selected sites and exerts a pathophysiological effect in AD, including impairment of physiological functions, apoptosis, neuroinflammation, and neuronal loss [84-86]. Meanwhile, the frontal lobe’s NFT burden correlates with dementia severity [87]. Therefore, reducing Aβ and NFTs has become a primary therapeutic target for AD.

EA mitigates cognitive impairment by lessening Aβ deposition [47, 51-54, 56, 59, 61, 63, 67, 68, 70, 74, 88] or the content of APP [56, 57, 60, 70, 88]. Tang *et al*. and Cai *et al*. indicated that EA decreased beta-site APP cleaving enzyme 1 (BACE1), identified as the β-secretase and involved in Aβ generation [57, 88]. Apolipoprotein E (APOE) can interact with Aβ, promoting aggregation and deposition [89]. Cai *et al*. stated that EA decreased the expression of APOE and might help reduce the deposit of Aβ [88]. The glymphatic system is a highly organized cerebrospinal fluid (CSF) transport system, together with lymphatics in surrounding tissues, which participates in the export of excess interstitial fluid and proteins and several vital functions [90]. The glymphatic pathway assists the neuropil of toxic proteinaceous metabolites such as Aβ, and its dysfunction has been demonstrated in animal models of AD and patients with AD [91]. Astrocytic aquaporin-4 (AQP4) plays a crucial role in mediating CSF entry into the brain parenchyma, mixing with interstitial fluid (ISF), and facilitating Aβ clearance [91]. Liang *et al*. reported that EA improved the glymphatic system mainly by improving AQP4 polarity to reduce Aβ accumulation [67]. In the meantime, EA also decreased the content of total tau or phosphorylated tau [54, 58, 59, 62, 63, 66, 69]. Glycogen synthase kinase-3β (GSK-3β) is a significant tau kinase involved in the abnormal hyperphosphorylation of tau protein during AD and a critical effector of phosphatidylinositol 3-kinase (PI3K)/ protein kinase B (Akt) cellular signaling [92]. GSK-3β became inactive when it was phosphorylated at Ser9 by activated Akt, which was phosphorylated at Ser473 [93, 94]. Targeting at PI3K/Akt/GSK-3β pathway has a potential role in improving cognitive impairment in AD. Yu *et al*. found that EA at 2Hz, 30Hz, and 50Hz downregulated the protein expression of total GSK-3β; nevertheless, it increased the phosphorylated-GSK-3β (Ser9) content in the hippocampus [56]. Similarly, Xu *et al*. indicated EA enhanced the ratio of phosphorylated-Akt/Akt as well as phosphorylated- GSK-3β (Ser9)/GSK-3β [62]. However, Yu *et al*. focused on disordered epigenetic modification. They showed that EA upregulated the methylation levels of CpG islands in the GSK-3β gene promoter and promoted the content of DNA methyltransferase (DNMT)1, which resulted in decreasing GSK-3β transcription and expression [69].

### 3.3 Effect of EA on Neuroinflammation

Neuroinflammation is the inflammatory response in the CNS caused by various pathological injuries; pro-inflammatory cytokines, small-molecule messengers, chemokines, and reactive oxygen species produced by innate immune cells in the CNS, mainly referring to microglia and astrocytes are the significant characteristics [95]. The brain of AD patients was analyzed and compared with normal controls; the brain tissue of AD patients had a higher concentration of pro-inflammatory factors, such as tumor necrosis factor-α (TNF-α) or interleukin-6 (IL-6), microglia and astrocytes were significantly activated, and the activation degree of astrocytes was associated with cognitive impairment [32]. Neuroinflammation has a significant role in AD pathogenesis [96].

EA was an effective method to ameliorate neuroinflammation in AD models [48, 54, 58, 61, 64, 66, 67, 70, 88]. Explicitly speaking, except for EA can reduce pro-inflammatory cytokines and relieve the activation of microglia and astrocytes, acting on the neuroinflammation-related signaling pathways is more worthy of attention. Zhang *et al*. indicated that EA upregulated the content of peroxisome proliferator-activated receptor-γ (PPAR-γ), which was crosstalk with other signaling pathways incorporated in AD and moderated neuroinflammation [54]. Enhancement of PPAR-γ activity played a role in anti-neuroinflammation and improvement of cognition [97]. Besides, p38 mitogen-activated protein kinase (p38MAPK) is considered an essential regulator of inflammation in the MAPK pathway [98]. It is believed that inhibiting p38MAPK signaling ultimately attenuates neuroinflammation [99, 100]. EA also reduced the phosphorylation levels of p38MAPK against neuroinflammation and cognitive impairment [54]. Pyroptosis is an inflammatory type of regulatory cell death that occurs downstream of the inflammasome signaling pathway [101]. NOD-like receptor protein-3 (NLRP3) inflammasome-driven inflammation and NLRP3/caspase-1 axis are critical in AD pathogenesis [102, 103]. Li and Hou *et al*. showed that EA significantly inhibited the levels of NLRP3 inflammasome and improved the activation of the NLRP3/caspase-1 axis [58, 66]. Gut microbiota modulates cognitive behavior *via* the microbiota-gut-brain axis, and the imbalance of gut microbiota may be closely related to AD; one involved mechanism is promoting neuroinflammation [104, 105]. Jiang *et al*. reported that EA ameliorated neuroinflammation by balancing the gut microbiota [64].

### 3.4 Effect of EA on Neuronal Apoptosis and Neurogenesis

In the mitochondrial pathway, B-cell lymphoma 2 (BCL-2) family proteins serve as the key regulators of apoptosis by mediating the mitochondrial outer membrane permeabilization (MOMP), which is the crucial step in activating the downstream caspase cascade to execute apoptosis [106]. BCL-2-associated X protein (BAX) and BAK form the mitochondrial outer membrane (MOM)-embedded oligomeric pores, which execute MOMP, while BCL-2 proteins such as BCL-2 exert an antiapoptotic effect through interactions with BAX or BAK to maintain them in an inactive state [107]. Besides, apoptosis can be triggered by death receptors (DRs), including Fas, Trail receptor (TRAIL-R), and tumor necrosis factor receptor 1 (TNFR1) binding to their ligands, which is called the death receptor pathway [108]. Apoptosis is crucial for the progression and pathology of neurodegeneration in AD [109, 110]. It is reported that apoptosis particularly occurs in vulnerable brain areas such as the hippocampus, accounting for cognitive impairment [111, 112]. Acting on attenuating neuronal apoptosis by EA treatment could reverse cognitive impairment in AD [50, 51, 52, 58, 60]. Tang *et al*. found that EA lessened the c-Jun N-terminal kinases (JNK) signaling pathway, which was identified as a crucial element for the regulation of apoptosis signals and correlated with AD pathogenesis [60, 113]. Lin *et al*. indicated that EA increased the content of mature isoform brain-derived neurotrophic factor (m-BDNF)/tyrosine kinase B (TrkB) signaling, which stimulated neuronal differentiation and dendritic morphogenesis and decreased proneurotrophin isoform of BDNF (pro-BDNF)/p75 neurotrophin receptor (p75NTR) signaling that involved in apoptosis in the hippocampus [52, 55, 114, 115].

However, neurogenesis happens throughout life in the dentate gyrus (DG) of the hippocampus, called adult hippocampal neurogenesis (AHN) [116]. DG sends information to CA3 and CA1 through the trisynaptic circuit, thus playing a significant role in learning and memory [117]. Through human brain samples, AHN dropped sharply in DG in patients with AD [118]. Emerging evidence has indicated a decline in AHN represents an early critical event during AD [119, 120]. Improving neurogenesis can rescue memory deficits in an animal model of AD by miR-132 and vitamin D [121, 122]. Moreover, EA also enhanced neurogenesis [47, 74]. Especially, EA probably activated the medial septal and vertical limb of the diagonal band (MS/VDB)-DG cholinergic neural circuit to regulate the cholinergic neural signal and promote neurogenesis in the DG to improve the early pattern separation [74].

### 3.5 Effect of EA on Autophagy

Autophagy is an essential catabolic process that can clear abnormal protein aggregates, restore protein homeostasis, and is vital for neuronal health [123]. It has been demonstrated that the control of autophagy is impaired and closely linked with AD, and enhancing autophagy can reduce AD-like pathology and cognitive decline [124-127]. EA mitigated autophagy dysfunction to improve cognitive decline [51, 70, 72]. Transcription factor EB (TFEB) is a positive regulator for the autophagy-lysosomal pathway and is impaired in developing neurodegenerative diseases, including AD [128]. The mechanistic target of rapamycin kinase complex 1 (MTORC1) mediates the autophagy-inhibitory effect and can be activated through upstream kinases such as Akt and MAPK [129]. Zheng *et al*. proved EA elevated the content of TFEB by inhibiting the Akt-MAPK1-MTORC1 pathway and then promoting autophagy against cognitive impairment [70].

### 3.6 Effect of EA on Energy Metabolism

AD is an age-related metabolic, neurodegenerative disease, and cerebral glucose hypometabolism acts as an invariant biomarker in AD [130]. The decline in glucose metabolism correlates with synaptic density and function and thus can alter cognitive function [131]. Therapeutic intervention targeting cognitive deficits in energy metabolism will be an effective strategy. EA treatment effectively improved brain glucose metabolism in AD models [53, 59, 61, 62, 65, 72, 88]. Specifically, EA directly triggered glucose transporters (GLUTs) [53], activated adenosine monophosphate-activated protein kinase (AMPK) signaling [49, 53, 65], which plays a role in sensing general energy and integrating nutrient and hormonal signals in the brain and is dysregulated in AD [132]. The impaired insulin signaling pathway is an essential pathogenesis of AD, significantly affecting cognitive impairment [133]. Huang *et al*. indicated EA elevated the insulin receptors’ content to improve the insulin signaling pathway in the hippocampus [59]. Besides, abnormal mitochondrial dynamics were observed in AD patients and animal models [134, 135]. Briefly, dysfunctional mitochondria can repair themselves by fusion with other healthy mitochondria, allowing the mixture of its contents to severely damage mitochondria through fission to their elimination by mitophagy [136]. Shao *et al*. showed EA enhanced mitochondrial fusion as well as decreased mitochondrial fission to attenuate mitochondrial damage and cognitive deficits [72].

### 3.7 Effect of EA on Synaptic Plasticity

Circumstances or personal experiences influence the strength and efficiency of synaptic connections. This property is known as synaptic plasticity [137]. Long-term potentiation (LTP) and long-term depression (LTD) are the two main types of synaptic plasticity, and they may occur in excitatory or inhibitory synapses through presynaptic and postsynaptic mechanisms [138]. Moreover, LTP in the hippocampus is thought to underlie learning and memory [139]. Both Ca^2+^-permeable N-methyl-D-aspartate receptors (NMDARs) and α-amino-3-hydroxy-5-methyl-4-isoxazole propionic acid receptors (AMPARs) are required for the induction of synaptic plasticity [137]. Dysregulation of synaptic plasticity participates in many neurodegenerative brain disorders, including AD, of which Aβ oligomers can disrupt LTP and increase LTD [140, 141]. EA effectively modulates synaptic plasticity *via* different mechanisms and then ameliorates cognitive impairment [50, 66, 71, 88]. First, EA activated 3′,5′-cyclic AMP (cAMP)/protein kinase A (PKA)/cAMP response element binding protein (CREB) pathway, which played a vital role in mediating LTP in the hippocampus [71, 142]. Second, EA enhanced the content of NMDARs and AMPARs to improve synaptic plasticity [66, 71]. Third, EA targeted decreasing the expression of Orexin A (OxA) in the CSF [71]. OxA was correlated with reduced synaptic plasticity [143]. Forth, EA mended synaptic function probably due to inhibiting the Notch pathway [50]. However, the Notch pathway is an essential requirement for neuronal function and memory formation and is implicated in AD [144]. Further studies are needed to illuminate precisely.

### 3.8 Other Mechanisms

EA reversed the upregulation of astrocytic N-myc downstream-regulated gene 2 (NDRG2) [48]. NDRG2 might be involved in AD’s pathologies, such as the accumulation of Aβ, neuronal apoptosis, and abnormal tau phosphorylation [145]. PI3K/Akt pathway participates in AD’s pathogenesis, including forming Aβ, neuronal apoptosis, and synaptic plasticity, so activating the PI3K/Akt pathway may mitigate the progression of AD [146]. Shao *et al*. showed EA activated PI3K/Akt pathway to exert a neuroprotective effect [72]. Nevertheless, EA downregulated the expression of the phosphorylated-PI3K and phosphorylated-Akt [59]. In order to reconcile the contradictive results, it could be speculated partly because of the difference in the animal models between Al/D-gal induced aging Ostuka Long-Evans Tokushima Fatty (OLETF) rat models and the Aβ-induced mouse model. In their research, Huang *et al*. pointed out that other downstream molecules could also suppress the activation of the PI3K/Akt pathway [59]. More studies are required to elucidate the precise position of the PI3K/Akt pathway in AD. EA reduced the level of glutamate in the CSF [71], which may become a new direction for AD intervention because an excess of glutamatergic transmission can lead to excitotoxicity and act as a general pathogenic mechanism in AD [147].

## 4 PARKINSON’S DISEASE

PD is the second most common neurodegenerative disorder after AD, characterized by tremors and bradykinesia, and is a common neurologic disease [148, 149]. Its pathological features involve cytoplasmic inclusions: Lewy bodies (LB) or Lewy neurites (LN) formed by misfolded α-synuclein aggregations [150]. According to the TCM, the pathogenesis of PD is based on the kidney deficiency. It initially manifests as the deficiency of the liver and kidney, the deficiency of yin and blood, the malnutrition of tendons and channels, and the endogenous liver wind. It gradually develops into the obstruction of qi and blood in the meridian, the binding of phlegm and blood stasis, and the combination of deficiency and excess. It still belongs to the deficiency in origin and the excess in superficiality. The origin lies in the deficiency of qi, blood, yin, and yang, while the superficiality is represented by wind, fire, phlegm, and stasis.

PD includes motor and nonmotor symptoms; motor symptoms commonly refer to tremors, bradykinesia, and akinesia, while nonmotor symptoms consist of cognitive impairment, depression, anxiety, sleep disturbance, and dysautonomia [148]. Cognitive impairment is a vital non-motor symptom of PD, and dementia can affect over 80% of PD patients who survive for 20 years [151]. Cognitive impairment in PD is believed to be associated with neurodegenerative brain changes, which mainly include degeneration of neurotransmitter systems, neuropathology, and genetic variation [152]. Acetylcholine (ACh) is thought to play an essential role in the cognitive deficits of PD [151]. A study showed that in the prefrontal cortex of 44 patients with AD, reduced choline acetyltransferase (ChAT) activity was related to cognitive impairment, which synthesized the neurotransmitter ACh [153]. Animal models currently used in PD research include drug-induced PD, neurotoxic animal models, agrochemical-induced animal models, transgenic models, α-synuclein models, and viral-vector-mediated models. However, none of the existing models will be able to fully replicate the comprehensive range of clinical features outlined in PD [154]. Shen *et al*. used 6-OHDA, one of the neurotoxic drugs, and found the reasons why EA could ameliorate cognitive impairment assessed through the Y-maze in PD rats were possibly connected with protecting cholinergic neurons and elevating ChAT activity [155]. The acupoint combination was still GV20 and GV14. Due to the lack of more relevant studies, there is still a broad space for research on whether EA can improve cognitive dysfunction and the possible mechanisms involved.

## 5 VASCULAR COGNITIVE IMPAIRMENT

Vascular cognitive impairment (VCI) is a term that contains a range of cognitive deficits associated with cerebrovascular pathology, ranging from MCI to VaD [156]. VCI accounts for 15-30% of dementia cases, second only to AD. [157]. The central cerebrovascular lesions in VCI are ischemic infarction and white matter hyperintensities (WMHs) [158]. According to the Chinese medicine theory, the pathological location of VCI is in the brain, with lesions occurring in mysterious mansions. The closure of mysterious mansions in the brain and the dysfunction of the vitality movement are the basic pathogenesis. Internal injuries and accumulated damage, as well as the deficiency of essence and qi, are internal factors contributing to the onset of the disease. Although diseases occur in the five organs, their origin lies in the kidney. The blockage of turbidity and toxins and the closure of the mysterious mansion are also important pathological features. Due to no widely accepted criteria delimiting the neuropathological threshold of VCI, there are seven disorders to predict, including arteriolosclerosis, perivascular space dilation, leptomeningeal cerebral amyloid angiopathy, myelin loss, microinfarcts, lacunar infarcts, and large infarcts [158, 159]. The exact pathophysiological mechanisms underlying VCI are not entirely clarified [160]. However, evidence indicated that many conditions, including but not limited to neuroinflammation [161], oxidative stress [162], synaptic plasticity [163], and apoptosis [164], can all participate in the progression of VCI, which give us several targets for intervention.

In the studies included in this part, VCI models are roughly divided into three categories from a pathophysiological point of view: cerebral hypoperfusion, cerebral ischemia-reperfusion, and ischemic stroke model. From the modeling method, VCI models included surgical models and transgenic models [165]. A brief literature summary about EA improving cognitive dysfunction in the VCI model is presented in Table **2**. The surgical model is used in all the literature in this part, and most authors adopt middle cerebral artery occlusion (MCAO) to conduct focal cerebral ischemia. The cortex, hippocampus, and striatum are mainly included in the damaged areas [165]. The remaining models are global ischemic stroke: bilateral common carotid artery occlusion (BCCAO), 2-vessel occlusion (2-VO), and bilateral carotid artery stenosis (BCAS) [165, 166].

Combined with the understanding of VCI in TCM, the Du meridian still dominates the choice of acupoints. GV20, which has the function of awakening the brain and clearing the mind, is still the first choice in most research. As mentioned in the AD section, GV24 is also the basic acupoint for treating cognitive impairment. We can quickly conclude from Table **2** that GV20 combined with GV24 became the most common selection for the treatment of VCI, followed by GV20 combined with GV14. It is also consistent with the conclusion obtained in a systematic review: GV20 combined with GV24 or GV14 are two of the three most frequently used acupoint combinations [167]. As the previous part already suggests, GV29 and KI3 can also play a role in improving cognitive impairment.

### 5.1 Effect of EA on Cognitive Function in VCI Models

For most of the studies in this section, MWM is still the first choice for assessing cognitive function [168-189]. Similarly, some of these studies also used the new object recognition [170, 187], step-down passive experiment [186, 190, 191], Y-maze test [192], and passive avoidance experiment [168] to evaluate cognitive dysfunction in various aspects, which is known as a classical method to evaluate non-declarative (conceptual) connecting memory in small animals [76]. Nonetheless, EA is an effective method to alleviate cognitive dysfunction in VCI models. The specific mechanisms will be discussed in the following.

### 5.2 Effect of EA on Synaptic Plasticity

Dendritic spines are small, thin, specialized projections of neuronal dendrites that mainly receive most of the excitatory synaptic input; thus, functional and structural changes in dendritic spines play a crucial role in modulating synaptic plasticity [193]. Cytoskeleton structures (actin), cytoskeleton proteins, cell membrane receptors (NMDARs, AMPARs, and metabotropic receptors), small GTPase and associated proteins, post-synaptic density (PSD) region, microRNA, mRNA binding proteins, transcription factors, extra-cellular matrix, and adhesion molecules are the main components of dendritic spines [194]. The actin cytoskeleton and PSD are the two major structural elements of the spines [195]. Actin primarily constitutes the cytoskeleton of the spine and is a major factor in the maintenance of spine structure [195]. Dendritic spines have been proposed to be a cellular basis of learning and memory [196]. Spine enlargement is correlated with LTP, and shrinkage represents LTD [197, 198]. The Rho family of small GTPases regulates spine morphogenesis by altering the activity of specific actin-binding proteins to promote or inhibit the polymerization of actin filaments [199]. Ras homologous member A (RhoA), Ras-related C3 botulinum toxin substrate 1 (Rac1), and cell division cycle 42 (Cdc42), three members of the Rho family of small GTPases, exert mostly [199]. Nonetheless, RhoA inhibits dendritic spines' growth and/or stability; Rac1 and Cdc42 promote [199]. PSD, which consists of a dense matrix of proteins located beneath the synaptic membrane, acts as a scaffolding platform for signaling networks, including NMDARs, AMPARs, and signaling molecules [195, 200]. The postsynaptic density-95 protein (PSD-95) is the main scaffold protein within the PSD of the dendritic spines and thus can reflect synaptic function, synaptic plasticity, and cognitive level [201, 202].

Ca^2+^ is probably the most crucial molecule in regulating the structure and function of synapses, dendritic excitability, and information storage through the neuronal circuit [203]. Ca^2+^ transforms the initial signaling by Ca^2+^-binding proteins. Then, Calmodulin (CaM) can transmit to other signaling proteins, such as Ca^2+^/CaM-dependent kinases (CaMKs), to play a crucial part during the induction of LTP [203]. CaMKs include CaMKI, CaMKII, CaMKIII, and CaMKIV, which trigger signaling activity by phosphorylating target proteins’ serine/threonine residues [204]. Among CaMKs, CaMKII participates in modulating the essential aspects of synaptic functions related to learning and memory, while CaMKIV phosphorylates the Ser-133 residue of CREB and activates memory-related transcriptional activity [204]. CREB-dependent gene expression is believed to be a crucial step in long-term synaptic plasticity and encoding long-lasting memories [205, 206]. CREB further alters the expression of BDNF [207], which activates signaling pathways by TrkB to regulate synaptic transmission and LTP in the hippocampus and other regions involved in presynaptic mechanisms, postsynaptic mechanisms, and dendritic spine morphology [208-210]. Meanwhile, BDNF level is significantly lower in patients with VCI [211].

EA improved synaptic plasticity in many aspects. First, EA enhanced dendritic spine plasticity [172, 175, 183]. Specifically, EA elevated the content of Cdc42, Rac1, and F-actin, decreasing the RhoA [175]. In the meantime, the density of dendritic spines, which is correlated with LTP [194], was increased after EA treatment [183]. In addition, EA modulated the expression of MicroRNAs (miRNAs) such as miR132 and miR134, increasing the expression of miR132 [172] while reducing miR134 [183]. miR132 repressed the level of p250GAP, a Rho family GTPase activating protein, to regulate spine size and density and improve synaptic plasticity [212]. miR134 inhibits LIM domain kinase 1 (LIMK1) synthesis, which regulates the actin cytoskeleton and spine expansion [213]. Second, EA increased the content of NMDARs, phosphorylated-NMDARs, AMPARs, and phosphorylated-AMPARs [170, 186, 187]. NMDARs and AMPARs are phosphorylated by many synapse-enriched protein kinases, including PKA, protein kinase C (PKC), CaMKII, non-receptor tyrosine kinases (NRTK), *etc*. [214]. Synaptic plasticity, including LTP and LTD, can be regulated by the phosphorylation of NMDARs and AMPARs [214]. Wang *et al*. pointed out that EA elevated the phosphorylation levels of AMPARs and NMDARs by inhibiting the 5-HT1A receptors, reducing PKA activity in the hippocampus [186]. Third, EA activated the BDNF/TrkB signaling to improve synaptic plasticity [177, 187]. However, Zheng *et al*. and Duan *et al*. reported EA increased the content of BDNF only, and they did not detect the change in TrkB [172, 189]. Fourth, studies reported the effect of EA in increasing the content of Ca^2+^ [186], CaMKII [170, 187], CaMKIV [190], and phosphorylated-CREB [172, 190], where Zhang *et al*. found EA directly activated the CaM/CaMKIV/CREB signaling in the hippocampus [190]. Fifth, EA promoted the expression of PSD-95 [177, 187, 191] and the number of synapses [177, 191]. Besides, EA downregulated the expression of Janus-activated kinase 2 (JAK2)/signal transducer and activator of transcription 3 (STAT3), which was associated with cognitive dysfunction after stroke [191]. Therefore, EA improves cognitive dysfunction by acting on multiple aspects of synaptic plasticity in VCI animal models. The mechanisms and signaling pathways involved are more complex and variable than EA, improving synaptic plasticity in AD animal models. It’s worth noting that EA increased the neurotrophin-4/5, which is bound to TrkB and phosphorylated CREB [168]. It was thought that their roles might be similar to BDNF to a certain extent [215]. Similarly, EA increased the content of nerve growth factor (NGF) in the hippocampal CA3 area [189]. NGF, as a neurotrophic factor, can also regulate synaptic plasticity and mediate neuroprotective effects [216, 217].

### 5.3 Effect of EA on Neuronal Apoptosis and Autophagy

Apoptosis is one of the major pathogenic factors involved in neuronal degeneration and axonal and white matter injury of VCI [218]. Unlike apoptosis, whether autophagy plays a beneficial or detrimental role in cerebral ischemic injury has not been clarified [219-222]. The autophagy’s time and levels may be the potential elements in determining the exact role [223].

EA was an effective method to reverse neuronal apoptosis [169, 173, 174, 179, 180] by increasing the content of BCL-2 and reducing the level of BAX or Fas. Among these researches, EA restrained the activation of nuclear factor-κB (NF-κB) signaling mediated neuronal cell apoptosis from ameliorating cognitive impairment [173]. For the VCI animal model, EA enhanced the level of autophagy against cognitive impairment [180, 188]. Wang *et al*. reported that EA reduced the expression level of the transcription factor p53 [180]. Although p53 is related to apoptosis, it stimulates antioxidant pathways and autophagy to promote cell survival [224]. EA also altered melatonin-mediated mitophagy to release a neuroprotective effect [188].

### 5.4 Effect of EA on Neuroinflammation

As aforementioned in AD, EA initially reduced pro-inflammatory cytokines and relieved microglia activation [171, 182, 188, 192]. EA also alleviated the NLRP3 pathway in VCI rats [188]. However, Han *et al*. found that IL-6 was increased after EA treatment [171]. It seems contradictory because IL-6 is upregulated whenever neuroinflammation is expected [225]. IL-6 in cerebral ischemic injury is controversial and needs further explanation [226-228]. The JAK2/STAT3 pathway is related to neuroinflammation, and its dysfunction has been observed in different brain disorders [229]. EA’s role in facilitating neuroinflammation was linked to downregulating the expression of the JAK2/STAT3 pathway [171]. Likewise, MAPK/extracellular signal-regulated kinase (ERK) also participated in neuroinflammation during the development of neurodegenerative diseases and altered memory [230]. Yang *et al*. reported that EA attenuated neuroinflammation by enhancing ERK activation [192]. Moreover, Purine receptors are a relatively novel target for relieving neuroinflammation by EA. They can be divided into adenosine (P1) and ATP (P2) receptors. And P2 receptors are further classified into two categories: ion channeltype P2X receptor (P2XR) and G protein-coupled metabotropic P2Y receptor (P2YR) [231]. P2X7 receptor (P2X7R) leads to microglia activation and the release of large amounts of pro-inflammatory factors when activated [232]. While the P2Y1 receptor (P2Y1R) also mediates neuroinflammation by releasing pro-inflammatory factors when activated by ATP [233]. Huang *et al*. showed that EA exerted its anti-inflammatory effect by inhibiting the P2X7R and P2Y1R on the astroglia and microglia [182]. The blood-brain barrier (BBB) is a barrier to the entry of microorganisms, toxins, bioactive substances, and a variety of substances, including drugs, into the brain [234]. Its disruption is often related to neuroinflammation [235]. Matrix metalloproteinase (MMP)s plays an essential role in the breakdown of the BBB and neuroinflammation [236]. Lin *et al*. suggested that EA reduced the expression of MMP-2 and MMP-9 proteins in the hippocampus and improved vascular disruption, thus mitigating cognitive impairment [176].

### 5.5 Effect of EA on Oxidative Stress

Oxidative stress is a phenomenon that results from the uncontrolled production of active substances, primarily reactive oxygen/nitrogen species (ROS/RNS). The main characteristic of these molecules is their high reactivity to biological components, lipids, proteins, nucleic acids, or carbohydrates [237]. ROS sources can be divided into enzymatic and non-enzymatic, as a by-product from oxidative phosphorylation occurring in the mitochondria and generated by nicotinamide adenine dinucleotide phosphate (NADPH) oxidases (NOXs) [238]. Nitric oxide, one of the RNS, is produced by nitric oxide synthases (NOS), which include endothelial NOS (eNOS), neuronal NOS (nNOS), and inducible NOS (iNOS) [239]. Alleviating oxidative stress by EA treatment is a valid method to improve VCI. EA enhanced the level of antioxidant enzyme systems such as superoxide dismutase (SOD) and catalase (CAT) and weakened the activation of ROS by reducing the content of NOXs, eNOS, and iNOS [169, 188]. Besides, EA upregulated the expression of miR-137 and thus led to the inhibition of NOX4 expression to regulate oxidative stress [169].

### 5.6 Other Mechanisms

Myelin, formed by oligodendrocytes (OLs) in the CNS, is the multilayer gelatinous membrane that surrounds the axon and is essential for electrical conductivity [240]. OLs and myelin are believed to be necessary for learning and memory [241, 242]. OLs derive from a lineage-restricted proliferative pool of OL precursor cells (OPCs), which can rapidly divide, differentiate, and respond to environmental variations [240, 241]. Ahn *et al*. suggested that EA promoted the recovery of cognitive impairment following white matter injury *via* promoting oligodendrocyte regeneration [168].

Excitotoxicity refers to the rapid release and inhibition of the excitatory amino acid glutamate reuptake caused by energy exhaustion. It is identified and intensively studied in the ischemic brain [243]. NMDA receptor 2B (NR2B) subunits, one of the three major subunit types of NMDARs, can mediate excitotoxic signaling by binding to the death-associated protein kinase 1 (DAPK1) and phosphorylated. Downregulation of NR2B and glutamate by EA exerted a neuroprotective effect for cerebral ischemia/reperfusion injury [178]. Similarly, the Downregulation of NR2B by moxibustion also did [244]. However, NR2B also plays a vital role in inducing LTP [245]. Dai *et al*. showed EA improved synaptic plasticity by increasing the content of NR2B [170]. Other studies aforementioned of expanding the content of NMDARs in VCI animal models only examined the expression of NMDA receptor 1 (NR1) subunits. The acupoints selected in both studies were the same. The reasons for the inconsistency may include different treatment parameters and different animal models. It has been proved that treatment parameters may bring other therapeutic effects [246]. The animal models are the bilateral typical carotid occlusion *versus* middle cerebral artery occlusion and reperfusion after 2 hours. Although reperfusion is a critical stage after thrombolysis or thrombectomy, it can result in reperfusion injury, including excitotoxicity [247]. Reperfusion plays a dual role: beneficial in the acute phase but potentially harmful in the convalescent stage [247]. Based on the above analysis, this contradiction in NR2B can be partially explained. More research is needed to clarify the cause of this discrepancy further. What needs to be added is that EA potentially relieved excitotoxicity by reducing excitatory neurotransmitters by upregulating the adenosine A1 receptors [179].

Advances in medical imaging techniques have enabled researchers to directly evaluate the changes in brain function *in vivo* under neurological diseases. Resting-state functional magnetic resonance imaging (rs-fMRI) is a technique of functional imaging to study and assess the connectivity between brain regions [248]. Wen *et al*. found that EA increased the amplitude of low-frequency fluctuations (ALFF) of cognition-related brain regions, which reflected the regional spontaneous brain activity [185]. Besides, proton magnetic resonance spectroscopy (1H-MRS) is a widely available technique for researching the biochemical milieu of the brain and can enhance the specificity of magnetic resonance imaging (MRI). He *et al*. reported that the neuroprotective effect of EA is probably *via* elevating the neurochemical metabolism of N-acetylaspartate (NAA) and choline (Cho) in the hippocampus and prefrontal cortex [184]. NAA and Cho are associated with cognitive function [249, 250].

Using proteomics and bioinformatics, Su *et al*. suggested that EA modulated cognitive impairment by targeting phosphatase and tensin homolog (Pten)/Akt pathway [181]. Proteomics identifies and quantifies overall proteins derived from cells, tissue, or organisms through many technologies [251]. It can bring us new insights into health and disease in neuroscience and push forward the frontiers of neuroscience research [252]. Study, development, or application of computational tools and methods are all included in bioinformatics to capture, store, visualize, and interpret medical or biological data [253]. Bioinformatics methods make it more efficient and influential in analyzing multi-omic data than simple methods [254]. It is highlighted that proteomics and bioinformatics have broad development space for EA research.

## 6 CHRONIC PAIN

Pain is defined by the International Association for the Study of Pain (IASP) as “an unpleasant sensory and emotional experience associated with actual or potential tissue damage, or described in terms of such damage” [255]. It is known that TCM pain can arise from qi impediment or blood stasis, as well as insufficiency of yin, yang, qi and blood, resulting in the loss of warmth and nourishment in the meridians, organs, and tissues. It is also associated with irregular qi movement or imbalances in yin and yang. Chronic pain, which usually lasts more than 12 weeks and is a common issue both in individual clinical practices and the healthcare system, can bring enormous global health burdens and affect more than 20% of people [256, 257]. There are three main categories of chronic pain: nociceptive pain, neuropathic pain, and nociplastic pain [258]. Both the two types of research included in this part for analysis using the neuropathic pain model: spared sciatic nerve injury [259] and trigeminal neuralgia induced by cobra venom [260], which are classic ways to mimic chronic pain. Depending on where the pain is located, *huantiao* (GB30) combined with *yanglingquan* (GB34) can relieve the pain in the subcostal region, and *shousanli* (LI10) combined with *quchi* (LI11) is an effective method to treat throat impediment and dentalgia. The principle of the treatment is mainly through circulating blood and relaxing tendons.

Cognitive and emotional factors have a vital influence on pain perception, and these factors will modulate intrinsic brain systems, such as opioid-related pain relief systems. Moreover, chronic pain can lead to cognitive deficits along the same circuitry [261]. It should be emphasized that the treatment of chronic pain is not limited to the relief of physical pain itself; biopsychosocial therapies, which focus more on increasing functional ability and satisfactory quality of life, are more prevalent [262]. A meta-analysis suggested that acupuncture affects the treatment of chronic pain and is a reasonable option for patients [263]. In the meantime, EA improved cognitive decline, possibly targeted at the transmembrane protein 126A (TMEM126A) and the excitatory amino acid transporters (SLC1A2/EAAT2) in rats with neuropathic pain [264]. Moreover, myristoylated alanine-rich C kinase substrate (marcks), p21-activated protein kinase (pak) 2, and acetyl coenzyme A acetyltransferase 1 (acat1) were related to the mechanisms of EA treating cognitive impairment caused by chronic pain [265]. Notably, these two articles used proteomics, transcriptomics, or bioinformatics to analyze potential therapeutic targets for EA. Like the proteomics concept, transcriptomics focuses on a complete set of genetic transcripts or RNA species transcribed in a specific cell type, tissue, or organism under specific physiological or pathological conditions [266]. These powerful technologies can bring us more possibilities.

## CONCLUSION AND FUTURE PERSPECTIVE

**Fig. (2):**
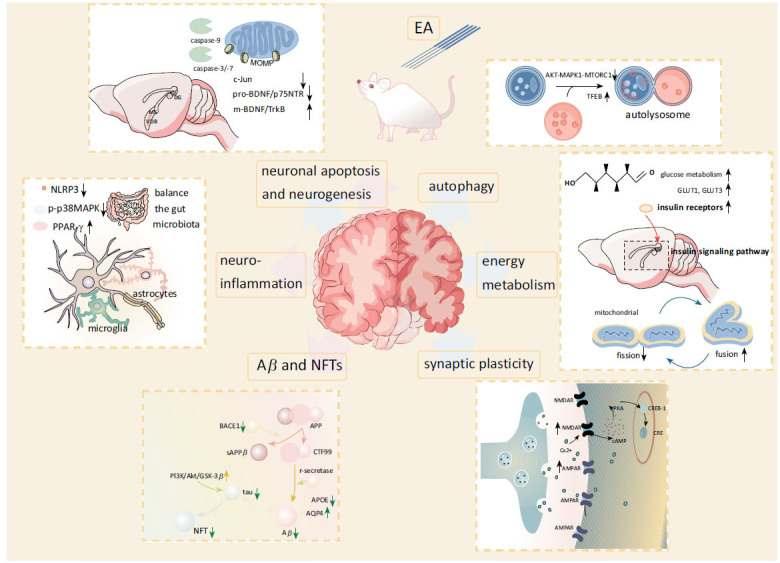
Main mechanisms of EA in AD. The blue arrows represent promotion, and the pink arrows represent inhibition. **Abbreviations**: Aβ: amyloid-β peptide; AD: Alzheimer's disease; AMPAR: α-amino-3-hydroxy-5-methyl-4-isoxazole propionic acid receptor; APOE: Apolipoprotein E; APP: amyloid-β protein precursor; Akt: protein kinase B; AQP4: aquaporin-4; BACE1: beta-site APP cleaving enzyme 1; cAMP: 3′,5′-cyclic AMP; CRE: cAMP response element; CREB: cAMP response element binding protein; CTFs: carboxyterminal fragments; EA: electroacupuncture; GLUT: glucose transporter; GSK-3β: glycogen synthase kinase-3β; MAPK: mitogen-activated protein kinase; m-BDNF: mature isoform brain-derived neurotrophic factor; MOMP: mitochondrial outer membrane permeabilization; MS: medial septal; mTOR: mechanistic target of rapamycin; MTORC1: mechanistic target of rapamycin kinase complex 1; NFT: neurofibrillary tangles; NLRP3: NOD-like receptor protein 3; NMDAR: N-methyl-D-aspartate receptor; p75NTR: p75 neurotrophin receptor; PI3K: phosphatidylinositol 3-kinase; PKA: protein kinase A; p-p38MAPK: phosphorylated-p38 mitogen-activated protein kinase; PPAR-γ: peroxisome proliferator-activated receptor-γ; pro-BDNF: proneurotrophin isoform of brain-derived neurotrophic factor; sAPP: soluble amyloid precursor proteins; TFEB: transcription factor EB; TrkB: tyrosine kinase B.

**Fig. (3):**
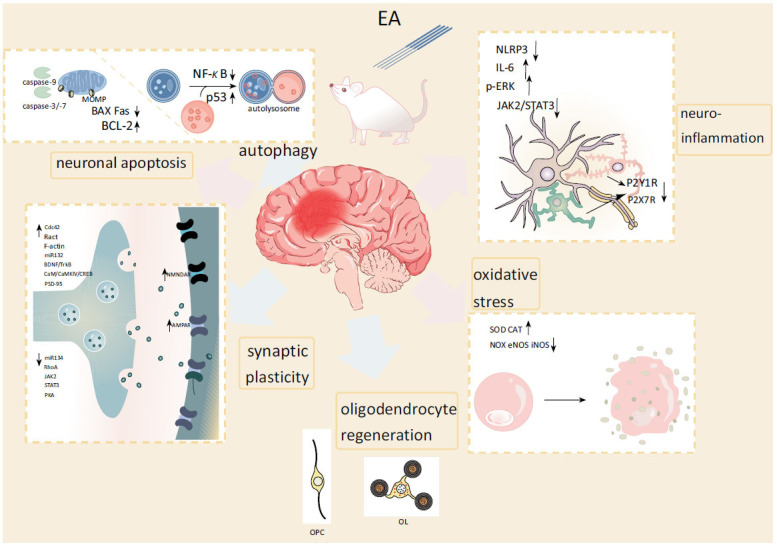
Main mechanisms of EA in VCI. The blue arrows represent promotion, and the pink arrows represent inhibition. **Abbreviations**: Akt: protein kinase B; AMPAR: α-amino-3-hydroxy-5-methyl-4-isoxazole propionic acid receptor; BAX: BCL-2-associated X protein; BCAS: bilateral stenosis of the common carotid artery; BCL-2: B-cell lymphoma 2; BDNF: brain-derived neurotrophic factor; CAT: catalase; CaM: Calmodulin; CaMK: Ca^2+^-calmodulin-dependent protein kinase; Cdc42: cell division cycle 42; eNOS: endothelial nitric oxide synthases; IL: interleukin; iNOS: inducible nitric oxide synthases; JAK2: Janus-activated kinase 2; MDA: malondialdehyde; miR: microRNA; MOMP: mitochondrial outer membrane permeabilization; NF-κB: nuclear factor kappa-B; NG2: neural/glial antigen 2; NLRP3: NOD-like receptor protein 3; NMDAR: N-methyl-D-aspartate receptor; NOX: nicotinamide adenine dinucleotide phosphate oxidases; OL: oligodendrocyte; OPC: oligodendrocyte precursor cell; p-ERK: phosphorylated-extracellular signal-regulated kinase; PKA: protein kinase A; PSD-95: postsynaptic density protein-95; P2XR: P2X receptor; P2YR: P2Y receptors; Rac1: Ras-related C3 botulinum toxin substrate 1; RhoA: Ras homologous member A; ROS: reactive oxygen species; SOD: superoxide dismutase; STAT3: signal transducer and activator of transcription 3.

This review of preclinical researches has summarized that EA improves cognitive impairment in neurological diseases, including AD (Fig. **2**), PD, VCI (Fig. **3**), and chronic pain, and induces potentially beneficial changes in molecular pathways. The mechanisms involved cover many aspects, such as synaptic plasticity, neuroinflammation, apoptosis, autophagy, *etc*. Also, EA’s treatment mechanisms are not entirely consistent for different diseases. However, these findings raise further questions.

First, acupoints are the loci where the qi of the zang-fu organs and meridians are transported to the body surface defined by TCM. Each point in a specific area can treat any disorder in nearby tissues and organs [267]. However, various therapeutic benefits are provided by stimulating different acupoints on the body’s surface [21]. It is crucial to select proper acupoints to treat individual diseases [268]. Therefore, even for the same illness, diverse acupoint selection may affect the specific mechanism of EA’s neuroprotective effect. Whether the different acupoint selection corresponds to the mechanisms of EA needs further study. Additionally, the therapeutic parameters of EA are also highly variable, which can mainly be summarized in four aspects: waveform, frequency, intensity, and time of duration. It has been reported that different waveforms and frequencies can affect the curative effect on the same disease [246, 269, 270]. The most appropriate choice of therapeutic parameters for a specific illness is still undiscovered. The study of the most suitable acupoints and treatment parameters is expected to be the focus of future research.

Moreover, although EA has shown excellent neuroprotective effects in preclinical research, there is still a long way to go before it can be used as an effective complementary treatment for cognitive dysfunction in neurological diseases. Only a few prospective clinical trials are currently involved in EA treatment for impaired cognition in stroke patients [271] and vascular cognitive impairment with no dementia patients [26]. Encouragingly, there has been a growing number of high-quality clinical studies on MA in recent years [272-276]. To some extent, it reflects that the clinical research on EA treatment of cognitive dysfunction in neurological diseases also has a broad exploration space.

Furthermore, TCM has been applied in China for thousands of years. However, due to the various ingredients and methods involved in the treatment of TCM, the effects on the human body are undoubtedly very complex and diverse. Therefore, it is difficult to effectively elucidate the mechanism behind TCM, which dramatically delays the pace of its modernization [277, 278]. Thanks to the development of omics and bioinformatics, there are more and more methods to use and study the potential targets of TCM treatment [277-282]. In particular, the rise of network pharmacology not only coincides with the holistic thinking of TCM but also effectively crosses the gap between Western medicine and TCM and promotes their synergistic development [279, 281]. Therefore, applying omics and bioinformatics in the future can bring great convenience for us to study the mechanism of EA against cognitive impairment in neurological diseases.

In addition, according to the Yellow Emperor’s Inner Canon (Huangdi Neijing), a doctor intervening in a disease before its occurrence is called “Shanggong.” Moreover, Invaluable Prescriptions for Emergencies (Beiji Qianjin Yaofang) also mentions the superior doctor prevents illness. However, most of the literature included in this review adopted establishing the disease model first and then processing EA therapy. In other words, EA treatment is started after symptoms appear. Meanwhile, it has been reported that EA pre-treatment can improve pain [282-284] and myocardial damage [285]. “Pre-treatment”, which is more in line with “the superior doctor”, may become another direction of EA’s future research in cognitive impairment of neurological diseases. Whereas EA has the potential for a neuroprotective effect, for high-risk people with cognitive dysfunction, EA treatment in advance will be one of the effective means to reduce the burden.

Significantly, ribonucleic acid binding proteins (RBPs), essential for ribonucleic acid (RNA) splicing, transport, translation, and stability, act on RNA targets and play the role of post-translational modification [286, 287]. Dysregulation of RBPs is currently linked to age-related diseases and neurodegenerative diseases [287, 288]. For instance, HuD, which regulates multiple AD candidate genes, promotes Aβ products through its upregulation; as for PD, some RBPs involve neurotoxicity and ROS formation procedures [288]. In this review, we mentioned that EA could exert neuroprotective effects by reducing the content of Aβ and alleviating oxidative stress. However, no study has reported whether EA can alter RBPs. Given that RBPs also participate in these pathophysiological processes, the role of EA in RBPs is expected to become one of the future research hotspots.

Finally, there is ample evidence for AD and VCI that EA can ameliorate cognitive impairment through various mechanisms. However, only a few studies focus on cognitive decline related to PD and chronic pain. Future research should pay close attention to elucidating EA's role and underlying mechanisms in PD and chronic pain-related cognitive impairment.

## Figures and Tables

**Fig. (1):**
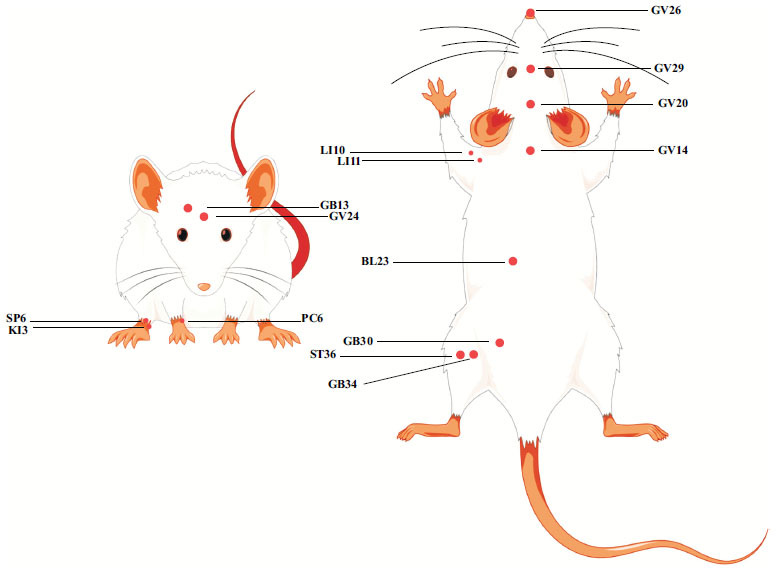
Schematic diagram of acupoints. **Abbreviations**: BL23: *shenshu*, GB13: *benshen*, GB30: *huantiao*, GB34: yanglingquan, GV14: *dazhui*, GV20: *baihui*, GV24: *shenting*, GV26: *shuigou*, GV29: *yintang*, KI3: *taixi*, LI10: *shousanli*, LI11: *quchi*, PC6: *neiguan*, SP6: *sanyinjiao*, ST36: *zusanli.*

**Table 1:** Summary of the therapeutic effect of EA on AD.

**Model**	**Acupoints**	**Therapeutic Parameters**	**Mechanisms**	**References**
APP/PS1 mice	GV20	**Waveform**: Do not mention frequency: 2/15 Hz **Intensity**: 1 mA **Duration**: 30 min daily, 5 days/week and 2 days rest for 4 weeks	NDRG2↓ GFAP↓	Wang *et al*. [48]
APP/PS1 mice	GV20	**Waveform**: Do not mention frequency: 2/15 Hz **Intensity**: 1 mA **Duration**: 30 min daily, 5 days/week and 2 days rest for 4 weeks	Aβ↓ BDNF↑ BrdU-positive cells↑	Li *et al*. [47]
SAMP8 mice	GV14, BL23	**Waveform**: Continuous wave frequency: 2 Hz **Intensity**: 1 mA, **Duration**: 20 min daily, 8 days, and 2 days rest for 30 days.	p-AMPK/AMPK↑	Dong *et al*. [49]
Intrahippocampally injected Aβ rat	GV20, BL23	**Waveform**: Continuous wave frequency: 20 Hz **Intensity**: ≤ 2 mA **Duration**: 30 min daily, 28 days with a rest every 7 days	BCL-2↑, BAX↓ Synapsin-1, Synaptophysin↑ Notch signaling pathway↓	Guo *et al*. [50]
APP/PS1 mice	GV20	**Waveform**: Disperse waves frequency: 1 and 20 Hz **Intensity**: Do not mention **Duration**: 30 min daily for 4 weeks	Aβ↓ BDNF/pro-BDNF↑ p-TrkB/TrkB↑ p75NTR↓	Lin *et al*. [52]
Intrahippocampally injected Aβ rat	GV20, BL23	**Waveform**: Do not mention frequency: 2 Hz **Intensity**: 2 mA **Duration**: 20 min daily, 5 days/week, and 2 days rest for 4 weeks	Aβ↓ p-tau↓ PPAR-γ↑ p-p38MAPK↓	Zhang *et al*. [54]
Intrahippocampally injected Aβ rat	GV20, BL23	**Waveform**: Continuous wave frequency: 20 Hz **Intensity**: ≤ 2 mA **Duration**: 30 min daily for 28 days with one rest day every 7 days	Aβ↓ cell apoptosis↓ LC3II/LC3I↑ Beclin-1↑	Guo *et al*. [51]
APP/PS1 mice	GV20	**Waveform**: Disperse waves frequency: 1 and 20 Hz **Intensity**: Do not mention **Duration**: 30 min daily, 5 days/week and 2 days rest for 4 weeks	Aβ↓ glucose metabolism↑ GLUT1, GLUT3↑ p-AMPK/AMPK↑ p-Akt/Akt↑ p-mTOR/mTOR↓	Liu *et al*. [53]
APP/PS1 mice	GV20	**Waveform**: Disperse waves frequency: 1 and 20 Hz **Intensity**: 1 mA **Duration**: 30 min daily for 4 weeks	NAA/Cr, Glu/Cr↑ BDNF↑ p-TrkB/TrkB↑	Lin *et al*. [55]
5XFAD mice	KI3	**Waveform**: Do not mention frequency: 2 Hz **Intensity**: 1 mA **Duration**: 15 min daily, three times per week for 2 weeks	APP, Aβ, APOE↓ glucose metabolism↑ CD11b, GFAP, COX2↓ HO-1, Transferrin↓ BAX↓ LTP↑ Synaptophysin, PSD-95↑ Synaptic ultrastructural degradation↓	Cai *et al*. [88]
APP/PS1 mice	GV20, GV26, GV29	For GV20 and GV29: **Waveform**: Do not mention frequency: 1 Hz **Intensity**: 1 mA **Duration**: 20 min each time, once every other day lasted for one month For GV26: fast pricking	APP, BACE1↓ p-PKA/PKA↑	Tang *et al*. [57]
Intrahippocampally injected Aβ rat	GV20, BL23	**Waveform**: Do not mention frequency: 2 Hz, 30 Hz, or 50 Hz **Intensity**: 1 mA **Duration**: 20 min daily, for 15 days with one rest day every 7 days	APP, Aβ↓ GSK-3β↓ p-GSK-3β (Ser9)↑ p-GSK-3β (Tyr216)↓ Synaptic ultrastructural degradation↓	Yu *et al*. [56]
APP/PS1 mice	GV20, GV26, GV29	For GV20 and GV29: **Waveform**: Do not mention frequency: 1 Hz **Intensity**: 1 mA **Duration**: 20 min each time, once every other day lasted for 4 weeks For GV26: fast pricking	APP↓ p-JNK/JNK↓ p-MKK4,7/MKK4,7↓ c-Jun↓	Tang *et al*. [60]
Intrahippocampally injected Aβ and intraperitoneally injected D-galactose rat	GV24, GB13	**Waveform**: Do not mention frequency: 30 Hz **Intensity**: 1 mA **Duration**: 30 min once daily for 28 days with one rest day every 6 days	Aβ↓ p-tau↓	Yang *et al*. [63]
APP/PS1 mice	GV20, GV26, GV29	For GV20 and GV29: **Waveform**: Do not mention frequency: 2 Hz **Intensity**: 1 mA **Duration**: 20 min each time, once every alternate day for 4 weeks For GV26: fast pricking	Aβ↓ glucose metabolism↑ Iba-1↓ IL-1β↓ IL-10↑	Xu *et al*. [61]
AlCl_3_-D-galactose-Ostuka Long-Evans Tokushima Fatty rat	GV20, ST36, BL23, SP6, PC6	**Waveform**: Sparse-dense waves frequency: 5 Hz **Intensity**: Do not mention **Duration**: 20 min daily for 3 weeks	Aβ↓ tau↓ FPG, FINS, C-P↓ p-IRS1, p-IRS2↑ IDE↑ p-PI3K↓ p-Akt↓ p-GSK-3β (Tyr216)↑	Huang *et al*. [59]
SAMP8 mice	GV20, ST36	**Waveform**: Do not mention frequency: 2 Hz or 10 Hz **Intensity**: 1 mA **Duration**: 30 min daily for 14 consecutive days	Aβ↓ tau↓ neuronal apoptosis↓ IL-1β, IL-6, IL-18, TNF-ɑ↓ NLRP3-Caspase1 axis↓	Hou *et al*. [58]
APP/PS1 mice	GV20, GV26, GV29	For GV20 and GV29: **Waveform**: Do not mention frequency: 2 Hz **Intensity**: 1 mA **Duration**: 20 min each time, once every other day for 28 days For GV26: fast pricking	p-tau/tau↓ glucose metabolism↑ p-Akt/Akt↑ p-GSK-3β (Ser9)/ GSK-3β↑	Xu *et al*. [62]
D-galactose-induced aging rat	GV20, BL23	**Waveform**: Continuous wave frequency: 50 Hz **Intensity**: 1 mA **Duration**: 20 min daily for 8 consecutive weeks	tau, p-tau↓ PHF-1↓ GSK-3β, p- GSK-3β (tyr216)↓ p-GSK-3β (Ser9)↑ GSK-3β mRNA↓ cytosine methylation in the promoter region of the GSK-3β gene↑ DNMT1↑	Yu *et al*. [69]
APP/PS1 mice	GV20, GV24	**Waveform**: Sparse and dense waves frequency: 1 and 20 Hz **Intensity**: 2V **Duration**: 30 min each time, 5 times a week, for 4 weeks	glucose metabolism↑ AMPK↑ PFKFB3, HK2, PKM2↑	Li *et al*. [65]
5XFAD mice	GB13, GV24	**Waveform**: Do not mention frequency: 2Hz **Intensity**: 0.3 mA **Duration**: 15 min each time, 5 times per week for 4 weeks	APP, Aβ, CTFs↓ Iba-1↓ LC3II, SQSTM1↓ LAMP1, CTSD↑ p-TFEB↓, TFEB↑ autolysosomes↑ recognition and degradation of autophagy substrates↑ Akt-MAPK1-MTORC1 pathway↓	Zheng *et al*. [70]
PS cDKO mice	GV20, GV24	**Waveform**: Do not mention frequency: 2 Hz **Intensity**: 1 mA **Duration**: 15 min each time, 5 consecutive days per week for 3 weeks	p-tau↓ LTP, NR1, NR2A, NR2B↑ NLRP3/Caspase-1 axis↓	Li *et al*. [66]
SAMP8 mice	GV20, GV29	**Waveform**: Sparse wave frequency: 2Hz **Intensity**: 2 V, 0.1 mA **Duration**: 15 min daily for 15 days	Delta-proteobacteria↑ Epsilon-proteobacteria↑ IL-1β, IL-6, TNF-α↓	Jiang *et al*. [64]
SAMP8 mice	GV20, GV26, GV29	For GV20 and GV29: **Waveform**: Continuous wave frequency: 2 Hz **Intensity**: 1mA **Duration**: 10 min each time, 5 consecutive days per week for 8 weeks For GV26: fast pricking	Aβ↓ the function of the glymphatic system↑ AQP4↑ GFAP↓	Liang *et al*. [67]
APP/PS1 mice	GV20, GV26, GV29	**Waveform**: Sparse wave frequency: 2 Hz **Intensity**: 2 V, 0.6 mA **Duration**: 20 min daily for 15 days	Aβ↓	Sun *et al*. [68]
Intrahippocampally injected Aβ mice	GV20, GV24, GB13	**Waveform**: Discrete wave frequency: 1∼20 Hz **Intensity**: 1 mA **Duration**: 15 min each time, 5 days per week for 3 consecutive weeks	Fis1, Fis1 mRNA↓ Mfn2, Mfn2 mRNA↑ Beclin1, LC3II↑ PI3K↑ p-Akt/Akt↑	Shao *et al*. [72]
SAMP8 mice	GV20, ST36	**Waveform**: Do not mention frequency: 10 Hz **Intensity**: 1 mA **Duration**: 30 min daily for 14 days	OxA↓ Glu↓ Nissl bodies of neurons↑ Synaptophysin, PSD-95↑ NR1, NR2A, AR2↑ cAMP/PKA/CREB pathway↑	Hou *et al*. [71]
5XFAD mice	GV20, GV24	**Waveform**: Do not mention frequency: 2/20 Hz **Intensity**: 1 mA **Duration**: 30 min each time, 5 times per week for 4 weeks	Aβ↓ the cholinergic system↑ activate MS/VDB-DG cholinergic neural circuit neurogenesis↑	Li *et al*. [74]

* **Abbreviations:** Aβ: amyloid-β peptide; AMPK: adenosine monophosphate-activated protein kinase; Akt: protein kinase B; APOE: Apolipoprotein E; APP: amyloid-β protein precursor; AQP4: aquaporin-4; AR: α-amino-3-hydroxy-5-methyl-4-isoxazole propionic acid receptor; BACE1: beta-site APP cleaving enzyme 1; BAX: BCL-2-associated X protein; BCL-2: B-cell lymphoma 2; BDNF: brain-derived neurotrophic factor; BL23: *shenshu*; BrdU: 5-bromo-2′-deoxyuridine; cAMP: 3′,5′-cyclic AMP; COX2: cyclooxygenase-2; C-P: C-peptide; Cr: creatine; CREB: cAMP response element binding protein; CTFs: carboxyterminal fragments; CTSD: cathepsin D; DG: dentate gyrus; DNMT1: DNA methyltransferase 1; Fis1: fission protein1; FINS: fasting insulin; FPG: fasting blood glucose; GB13: Benshen; GFAP: glial fibrillary acidic protein; Glu: glutamate; GLUTs: glucose transport proteins; GSK-3β: glycogen synthase kinase-3β; GV14: *dazhui*; GV20: *baihui*; GV24: *shenting*; GV26: *shuigou*; GV29: *yintang*; HK2: hexokinase II; HO-1: heme oxygenase 1; IDE: insulin-degrading enzyme; Iba-1: Ionized calcium-binding adapter molecule 1; IL: interleukin; IRS: insulin receptor substrate; JNK: c-Jun N-terminal kinases; KI3: *taixi*; LAMP1: lysosomal-associated membrane protein 1; LC3: microtubule-associated protein 1 light chain 3; LTP: long term potentiation; MAPK: mitogen-activated protein kinase; Mfn2: mitochondrial protein 2; MKK: mitogen-activated protein kinase kinase; MS: medial septal; mTOR: mechanistic target of rapamycin; MTORC1: mechanistic target of rapamycin kinase complex 1; NAA: N-acetylaspartate; NDRG2: N-myc downstream-regulated gene 2; NLRP3: NOD-like receptor protein 3; NR: N-methyl-D-aspartate receptor; OxA: Orexin A; p75NTR: p75 neurotrophin receptor; p-AMPK; phosphorylated-adenosine monophosphate-activated protein kinase; p-Akt: phosphorylated-protein kinase B; PC6: *neiguan*; PFKFB3: 6-phosphofructo-2-kinase/fructose-2,6-biphosphatase 3; p-GSK-3β: phosphorylated-glycogen synthase kinase-3β; PHF-1: paired helical filament 1; PI3K: phosphatidylinositol 3-kinase; p-PI3K: phosphorylated- phosphatidylinositol 3-kinase; p-IRS: phosphorylated-insulin receptor substrate; p-JNK: phosphorylated-c-Jun N-terminal kinases; PKA: protein kinase A; PKM2: pyruvate kinase M2; p-MKK: phosphorylated-mitogen-activated protein kinase kinase; p-mTOR: phosphorylated-mechanistic target of rapamycin; p-p38MAPK: phosphorylated-p38 mitogen-activated protein kinase; PPAR-γ: peroxisome proliferator-activated receptor-γ; p-PKA: phosphorylated-protein kinase A; pro-BDNF: proneurotrophin isoform of BDNF; PS cDKO: presenilin 1 and 2 conditional double knockout; PSD-95: postsynaptic density protein-95; p-tau: phosphorylated-tau; p-TFEB: phosphorylated-transcription factor EB; p-TrkB: phosphorylated-tyrosine kinase B; SAMP8: senescence-accelerated mouse prone 8; SP6: *sanyinjiao*; SQSTM1: sequestosome 1; ST36: *zusanli*; TFEB: transcription factor EB; TNF-α: tumor necrosis factor-α; TrkB: tyrosine kinase B; VDB: vertical limb of the diagonal band; ↑: upregulated; ↓: downregulated.

**Table 2:** Summary of the therapeutic effect of EA on VCI.

**Model**	**Acupoints**	**Therapeutic Parameters**	**Mechanisms**	**References**
2VO rat	GV14, GV20	**Waveform:** Do not mention frequency: 20 Hz **Intensity:** Do not mention **Duration:** 20 min daily for 7 days	LTP↑ dendritic spine density↑ p-CREB, BDNF↑ miR132↑, p250GAP↓	Zheng *et al*. [172]
BCAS mice	GV14, GV20	**Waveform:** Do not mention frequency: 20 Hz **Intensity:** 2V **Duration:** 20 min daily for 7 days	White matter injury↓ MBP↑ BrdU-positive cells↑ NG2, PDGFRα↓ CNPase↑ NT4/5, p-TrkB↑ p-CREB↑	Ahn *et al*. [168]
BCCAO rat	GV14, GV20	**Waveform:** Dilatational wave frequency: 2/15 Hz **Intensity:** 2 ± 1 mA **Duration:** 30 min each time, 5 times a week for 4 weeks	rCBF↑ IL-6↑ TNF-ɑ, IL-1β↓ JAK2↓	Han *et al*. [171]
BCCAO rat	GV14, GV20	**Waveform:** Do not mention frequency: 2/15 Hz **Intensity:** 1 mA **Duration:** 30 min daily for 4 weeks	NOX1, NOX2, NOX4↓ miR137↑ neuronal apoptosis↓ GFAP, eNOS, iNOS↓ ROS, MDA↓, SOD, CAT↑	Bi *et al*. [169]
2VO rat	GV20, GV24	**Waveform:** Sparse-dense waves frequency: 2/20 Hz **Intensity:** 6 V, 1-3 mA **Duration:** 30 min each time, 5 times a week for 4 weeks	LTP↑ excitatory synaptic transmission dynamics↑ p-AR1, p-NR2B↑ CaMKII, p-CaMKII↑	Dai *et al*. [170]
MCAO/R rat	GV20, GV24	**Waveform:** Disperse waves frequency: 1 and 20 Hz **Intensity:** Do not mention **Duration:** 30 min daily for 10 days	Infarction volume↓ neuronal apoptosis↓ p-IκB/IκB, NF-κB p65↓ BAX, Fas↓	Feng *et al*. [173]
MCAO/R rat	GV20, GV24	**Waveform:** Disperse waves frequency: 1-20 Hz **Intensity:** 6 mA **Duration:** 30 min daily for 14 days	Improve neurological deficit scores neuronal apoptosis↓ BAX↓, BCL-2↑	Liu *et al*. [174]
MCAO/R rat	GV20, GV24	**Waveform:** Disperse waves frequency: 1 and 20 Hz **Intensity:** do not mention **Duration:** 30 min daily for 7 days	Infarction volume↓ Cdc42, Rac1↑, RhoA↓ F-actin↑ the density of dendritic spines↑	Lin *et al*. [175]
BCCAO Mongolian gerbils	GV20, KI3	**Waveform:** Do not mention frequency: 2 Hz **Intensity:** 1mA **Duration:** 20 min each time, every other day for 7 days	Iba-1, TLR4, TNF-ɑ↓ p-ERK/ERK↑ glucose metabolism↑	Yang *et al*. [192]
MCAO/R rat	GV20, GV24	**Waveform:** Do not mention frequency: 1 and 20 Hz **Intensity:** 1-3 mA **Duration:** 30 min daily for 7 days	Improve neurological deficit scores infarction volume↓ vascular disruption↓ MMP-2, MMP-9↓	Lin *et al*. [176]
MCAO/R rat	GV20, GV24	**Waveform:** Disperse wave frequency: 1 and 20 Hz **Intensity:** Do not mention **Duration:** 30 min daily for 7 days	Infarction volume↓ CaM↑ CaMKIV, p-CaMKIV↑ CREB, p-CREB↑	Zhang *et al*. [190]
MCAO/R rat	GV20, GV24	**Waveform:** Disperse wave frequency: 2/10 Hz **Intensity:** 1-3 mA **Duration:** 30 min daily for 7 days	Improve neurological deficit scores infarction volume↓ BDNF, TrkB↑ PSD-95↑	Lin *et al*. [177]
MCAO rat	GV14, GV20	**Waveform:** Continuous wave frequency: 5 or 50 Hz **Intensity:** 1.5-3 V, 1-2 mA **Duration:** 15 min daily for 14 days	BDNF↑ NGF↑	Duan *et al*. [189]
MCAO/R rat	GV20, GV24	**Waveform:** Do not mention frequency: 20 Hz **Intensity:** Do not mention **Duration:** 30 min daily for 7 days	Infarction volume↓ NR2A↑, NR2B↓ Ca^2+^↓	Zhang *et al*. [178]
MCAO/R rat	GV20, GV24	**Waveform:** Dilatational wave frequency: 1-20 Hz **Intensity:** 6 V, 2 mA **Duration:** 30 min daily for 8 days	Improve neurological deficit scores infarction volume↓ cell apoptosis↓ p53↓, Beclin-1↑ PI3K↑, mTOR↑, p-Akt↑	Wang *et al*. [180]
MCAO/R rat	GV20	**Waveform:** Disperse wave frequency: 2/15 Hz **Intensity:** 1 mA **Duration:** 30 min once	Adenosine A1 receptor↑ BCL-2/BAX↑	Shi *et al*. [179]
MCAO/R rat	GV20, GV24	**Waveform:** Dilatational wave frequency: 1-20 Hz **Intensity:** 1-3 mA **Duration:** 30 min daily for 14 days	Improve neurological deficit scores infarction volume↓ Pak4, Akt3, Efnb2↑	Su *et al*. [181]
MCAO/R rat	GV20, GV24	**Waveform:** Dilatational wave frequency: 2/20 Hz **Intensity:** 6 V, 0.2 mA **Duration:** 30 min daily for 7 days	Improve neurological deficit scores infarction volume↓ ED1^+^, GFAP^+^ cells↓ IL-1β↓, IL-10↑ P2X7R, P2Y1R↓	Huang *et al*. [182]
MCAO/R rat	GV20, GV24	**Waveform:** Dilatational wave frequency: 1-20 Hz **Intensity:** 6 V, 0.2 mA **Duration:** 30 min daily for 14 days	Infarction volume↓ the density of dendritic spines↑ number of synapses↑ p-LIMK1/LIMK1↑ miR134↓	Liu *et al*. [183]
MCAO/R rat	GV20, GV24	**Waveform:** Dilatational wave frequency: 1-20 Hz **Intensity:** 6 V, 1 mA **Duration:** 30 min daily for 7 days	NAA/Cr↑ Cho/Cr↑ Glu/Cr↑	He *et al*. [184]
MCAO/R rat	GV20, GV24	**Waveform:** Dilatational wave frequency: 1-20 Hz **Intensity:** 0.2 mA **Duration:** 30 min daily for 14 days	Infarction volume↓ ALFF↑	Wen *et al*. [185]
MCAO/R rat	GV20, GV24	**Waveform:** Condensation and rarefaction waves frequency: 1-20 Hz **Intensity:** 0.2 mA **Duration:** 30 min daily for 14 days	Improve neurological deficit scores PSD-95, synaptophysin↑ number of synapses↑ p-JAK2/JAK2↓ p-STAT3/STAT3↓	Xie *et al*. [191]
MCAO/R rat	GV20, GV24	**Waveform:** dilatational wave frequency: 2/20 Hz **Intensity:** 6V, 0.2mA **Duration:** 30 min daily for 14 days	Improve neurological deficit scores infarction volume↓ 5-HT1AR↓ p-NR1/NR, p-AR1/AR↑ PKA, Ca^2+^↑	Wang *et al*. [186]
MCAO/R rat	GV20, GV29	**Waveform:** Continuous wave frequency: 2 Hz **Intensity:** 1 mA **Duration:** 10 min daily for 14 days	BDNF, TrkB↑ NR1, AR, GABA_^A^_R↑ CaMKII↑ PSD-95, NeuN↑	Zheng *et al*. [187]
MCAO/R rat	GV20, GV24	**Waveform:** Dilatational wave frequency: 4 and 20 Hz **Intensity:** 2 V, 0.5 mA **Duration:** 30 min daily for 7 days	infarction volume↓ AANAT↑ LC3II/LC3I, Beclin-1↑ Parkin, PINK1↑ ROS↓ NLRP3/Caspase1 axis↓ Iba-1^+^ cells↓	Zhong *et al*. [188]

* **Abbreviations:** AANAT: aralkylamine N-acetyltransferase; Akt: protein kinase B; AR: α-amino-3-hydroxy-5-methyl-4-isoxazole propionic acid receptor; BAX: BCL-2-associated X protein; BCAS: bilateral stenosis of the common carotid artery; BCCAO: bilateral common carotid artery occlusion; BCL-2: B-cell lymphoma 2; BDNF: brain-derived neurotrophic factor; BrdU: 5-bromo-2′-deoxyuridine; CAT: catalase; CaM: Calmodulin; CaMK: Ca^2+^-calmodulin-dependent protein kinase; Cdc42: cell division cycle 42; CNPase: 2,3-cyclic nucleotide-3-phosphodiesterase; Cr: creatine; Efnb2: ephrin-B2; eNOS: endothelial nitric oxide synthases; ERK: extracellular signal-regulated kinase; GABA_A_R: γ-amino-butyric acid type A receptor; GFAP: glial fibrillary acidic protein; Glu: glutamate; GV14: *dazhui*; GV20: *baihui*; GV24: *shenting*; GV29: *yintang*; Iba-1: Ionized calcium-binding adapter molecule 1; IL: interleukin; iNOS: inducible nitric oxide synthases; IκB: inhibitor proteins of NF-κB; JAK2: Janus-activated kinase 2; KI3: *taixi*; LC3: microtubule-associated protein 1 light chain 3; LIMK1: LIM domain kinase 1; MBP: myelin basic protein; MCAO: middle cerebral artery occlusion; MCAO/R: middle cerebral artery occlusion and reperfusion; MDA: malondialdehyde; miR: microRNA; MMP: Matrix metalloproteinase; mTOR: mechanistic target of rapamycin; NAA: N-acetylaspartate; NeuN: neuron-specific nuclear protein; NF-κB: nuclear factor kappa-B; NG2: neural/glial antigen 2; NGF: nerve growth factor; NLRP3: NOD-like receptor protein 3; NOX: nicotinamide adenine dinucleotide phosphate oxidases; NR: N-methyl-D-aspartate receptor; NT4/5: neurotrophin-4/5; LTP: long term potentiation; p-Akt: phosphorylated-protein kinase B; Pak: p21-activated protein kinase; p-AR: phosphorylated-α-amino-3-hydroxy-5-methyl-4-isoxazole propionic acid receptor; p-CaMK: phosphorylated-Ca^2+^-calmodulin-dependent protein kinase; p-CREB: phosphorylated-cAMP response element binding protein; PDGFRα: platelet-derived growth factor receptor-α; p-ERK: phosphorylated-extracellular signal-regulated kinase; PI3K: phosphatidylinositol 3-kinase; p-IκB: phosphorylated-inhibitor proteins of NF-κB; p-JAK2:phosphorylated-Janus-activated kinase 2; PKA: protein kinase A; p-LIMK1: phosphorylated-LIM domain kinase 1; PINK1: PTEN induced putative kinase 1; p-NR: phosphorylated-N-methyl-D-aspartate receptor; PSD-95: postsynaptic density protein-95; p-STAT3: phosphorylated-signal transducer and activator of transcription 3; p-TrkB: phosphorylated-tyrosine kinase B; P2XR: P2X receptor; P2YR: P2Y receptors; Rac1: Ras-related C3 botulinum toxin substrate 1; rCBF: regional cerebral blood flow; RhoA: Ras homologous member A; ROS: reactive oxygen species; SOD: superoxide dismutase; STAT3: signal transducer and activator of transcription 3; TNF-α: tumor necrosis factor-ɑ; TLR4: toll-like receptor 4; 2VO: 2-vessel occlusion; 5-HT1AR: serotonin (1A) receptor; ↑: upregulated; ↓: downregulated.

## LIST OF ABBREVIATIONS

ACh: Acetylcholine
AD: Alzheimer’s Disease
AHN: Adult Hippocampal Neurogenesis
APP: Amyloid-β Protein Precursor
BBB: Blood-brain Barrier
CNS: Central Nervous System
CTFs: Carboxyterminal Fragments
IL-6: Interleukin-6
iNOS: Inducible NOS
LTD: Long-term Depression
LTP: Long-term Potentiation
MA: Manual Acupuncture
MCI: Mild Cognitive Impairment
MOM: Mitochondrial Outer Membrane
MRI: Magnetic Resonance Imaging
NOS: Nitric Oxide Synthases
OLs: Oligodendrocytes
PD: Parkinson's Disease
PSD: Post-synaptic Density
sAPP: Soluble Amyloid Precursor Proteins
TCM: Traditional Chinese Medicine
TFEB: Transcription Factor EB
TNF-α: Tumor Necrosis Factor-α
VaD: Vascular Dementia
VCI: Vascular Cognitive Impairment
